# Raman Spectroscopy Analysis of the Biochemical Characteristics of Molecules Associated with the Malignant Transformation of Gastric Mucosa

**DOI:** 10.1371/journal.pone.0093906

**Published:** 2014-04-07

**Authors:** Yao Chen, Jianhua Dai, Xueqian Zhou, Yunjie Liu, Wei Zhang, Guiyong Peng

**Affiliations:** 1 Institute of Gastroenterology, Southwest Hospital, Third Military Medical University, Chongqing, China; 2 Key Laboratory of Multi-scale Manufacturing Technology, Chongqing Institute of Green and Intelligent Technology, Chinese Academy of Sciences, Chongqing, P. R. China; Taipei Medical University, Taiwan

## Abstract

**Objective:**

The purpose of this study was to comparatively analyze the signature Raman spectra of genomic DNA, nuclei, and tissue of normal gastric mucosa and gastric cancer and to investigate the biochemical transformation of molecules associated with gastric mucosa malignancy.

**Method:**

Genomic DNA, nuclei, and tissue from normal gastric mucosa and gastric cancer were analyzed by Raman spectroscopy.

**Results:**

1) The Raman spectrum of gastric cancer genomic DNA showed that two peaks appeared, one at approximately 1090 cm^-1^ with a higher intensity than the peak at 1050 cm^-1^ in the spectrum. Characteristic peaks appeared at 950 cm^-1^, 1010 cm^-1^, and 1100-1600 cm^-1^. 2) Using a hematoxylin and eosin (H&E)-stained section, the intensity of the characteristic peak of nucleic acids at 1085 cm^-1^ was increased and shifted to 1088 cm^-1^ in cancer cells. The relative intensity of the characteristic peaks of nucleoproteins at 755 cm^-1^ and 1607 cm^-1^ was significantly increased in cancer cells compared with normal cells. 3) Compared with normal tissues, the peak representing PO^2-^ symmetric stretching vibration shifted from 1088 cm^-1^ to 1083 cm^-1^ in cancer tissue, and the characteristic peak for collagen at 938 cm^-1^ shifted to 944 cm^-1^. In addition, an extra characteristic peak indicating C = C stretching vibration appeared at 1379 cm^-1^ in the lipid spectrum in cancer tissue.

**Conclusions:**

The position, intensity, and shape of peaks in the Raman spectra of DNA, nuclei, and tissue from gastric cancer were significantly different compared with those of normal cells. These results indicate that the DNA phosphate backbone becomes unstable in cancer cells and might be broken; the relative content of histones is increased and stable; the relative collagen content is reduced, facilitating cancer cell metastasis; and the relative content of unsaturated fatty acids is increased, increasing the mobility of the plasma membrane of cancer cells.

## Introduction

Gastric cancer is one of the most commonly occurring malignant tumors and seriously threatens human health. Early diagnosis and treatment are globally recognized as the most effective approach to improving survival, and early diagnosis is particularly crucial. Diseases initiate from changes in tissue and intracellular structure and composition. The characterization of tissues and cellular structure and composition at different stage of gastric cancer development will not only advance our understanding of gastric cancer initiation but also improve early diagnosis.

Raman spectrometry is an ideal approach for analyzing the spatial structure of matter. A Raman spectrum is an emission spectrum generated by high-frequency, inelastic scattering of light resulting from the response of a molecule to incident rays. Every macromolecule has a unique signature Raman spectrum. When normal cells or tissues undergo a process of disease initiation, the structural conformation and relative content of macromolecules that constitute a cell and tissue change. For example, the phosphate backbone of nucleic acids changes, hydrogen bonds between protein molecules are damaged, and the lateral stacking force of lipid molecules is reduced. All of these changes contribute to corresponding unique changes in the Raman spectra of the molecules in cancer tissues. Thus, analysis of Raman spectra can reveal physiological, biochemical, and structural changes in cells and tissues and can characterize the biochemical transformation of molecules associated with malignancy [1]. In addition, the use of Raman spectrometry to analyze biomolecules has the advantages of requiring a small amount of sample, being fast and resistant to water interference, not causing damage to the tissue, and allowing for in situ detection. Thus, Raman spectrometry is widely used in medical fields. Its uses include the determination of the secondary structure of proteins and of the interactions between DNA and anti-cancer drugs, the diagnosis of damaged cells and tissue, and the analysis of patient bodily fluids, such as serum [2]–[12]. It has been reported that the sensitivity and specificity of using Raman spectrometry to diagnose gastric mucosal lesions in vivo are 85%–95% and 90%–98%, respectively [13]. Scientists now mainly focus on the differential comparison of Raman spectra, the establishment of diagnostic models and principles by combining Raman spectrometry and multivariate statistics, and distinguishing malignant versus benign tumors, pathological subtypes, degree of differentiation, and lymph node metastasis [1], [4]–[7], [10]. Raman spectrometry has not been utilized to its full potential to analyze the microstructure of molecules and the mechanisms and principles associated with malignancy of tissue and cells. [14] J.M.Hu and co-workers characterised gastric carcinoma cell in both cultured cells and mucosa tissues by confocal Raman microspectroscopy. Their results indicated that there were obvious spectral alterations associated with malignancy compared with normal ones, such as intensity of 1587 cm^-1^ decreased, peak shape of 1660 cm^-1^ changed. [5] Zhuang Z and co-workers analyzed raman spectrum of normal and malignant renal tissues and discovered that I_855 cm-1_/I_831 cm-1_ decreased obviously in tumor tissues. This suggest that more tyrosine conformation transform from “buried” to “exposed” and then structure of some protein tend to be instable with canceration).

We used Raman spectrometry to analyze genomic DNA, nuclei, and tissue from normal and malignant gastric mucosa and characterized the peaks in the spectra. Based on the vibration of chemical and functional groups, including C-C, PO^2-^, C = C, and phenyl groups, in corresponding macromolecules, such as DNA, RNA, proteins, lipids, and carotene, we investigated the changes in spatial structure and biochemical composition in mucosal tissue during cancer development. Our study provides a theoretical basis for understanding the tissue transformation during gastric cancer development from the perspective of molecular physiology and biochemistry and sheds new light on the early diagnosis of gastric cancer.

## Materials and Methods

### Ethics statement

This study was approved by the local Ethics Committee (Ethics Committee of Southwest Hospital). Prior to specimen collection, all patients have signed informed consent forms.

### Reagents and instruments

Reagents and instruments used include cell lysis buffer (Shen Neng Bo Cai), a genomic DNA extraction kit (Tian Gen), formaldehyde (Chongqing Chuan Dong), a homogenizer and an electronic balance (SARTORIOUS), a UV spectrophotometer (BIO-RAD), a transmission optical microscope (OLYMPUS), a confocal micro-Raman spectroscope (ThermoFisher, British Renishaw), an automatic balancing microcentrifuge (Beijing Medical), and a heated water tank (Shanghai Jinghong).

### Experimental methods

#### Specimen preparation

Tissue specimens were collected from Southwest Hospital, first affiliated hospital of Third Military Medical University. Prior to specimen collection, patients signed informed consent forms. Normal gastric mucosal biopsy specimens were collected during gastroscopy. Gastric cancer tissues were collected either from surgery or from biopsy during gastroscopy. All patients were definitely diagnosed as primary gastric adenocarcinoma and received no treatment before specimen collection. The cancerous lesions were located at fundus, antrum, angle, cardia, pylorus and body of stomach respectively among patients. After Raman spectrometry measures, all specimens were pathologically confirmed again and were all diagnosed as advanced gastric cancer. Tissue specimens of normal and cancer were collected from 15 health people and patients respectively, including 8 female and 22 male, average age 55.03±14.27 years. Normal tissues were collected from all age groups and from each part of stomach separately.

#### DNA solution preparation

One hundred milligrams of tissue was weighed using an electronic balance. The tissue was mixed with cell lysis buffer and homogenized with a homogenizer immersed in ice. The cell lysate was transferred to a centrifuge tube. Genomic DNA was extracted from the cell lysate according to the manufacturer's instructions for the genomic DNA extraction kit. Genomic DNA was eluted in 50 μl of Tris-EDTA (TE) buffer. Fifty microliters of DNA solution was prepared from the three types of samples. DNA concentration was measured using a UV spectrophotometer and converted to the amount of DNA per solution volume. The DNA concentration was 0.5/1000–2.7/1000.

#### H&E section preparation

Specimens were fixed with 10% formalin, embedded in paraffin, and sectioned at a thickness of 20 μm. The tissue sections were stained with H&E and observed under an optical microscope to confirm the tissue diagnosis. The tissue sections were then examined by confocal Raman spectroscopy.

#### Tissue preparation

Fresh biopsy specimens collected during gastroscopy from either gastric cancer or normal gastric mucosa were stored in 1.8-ml sterile vials kept on ice and transported to the Raman spectrometry laboratory (Raman spectrometry was performed within 1 h of tissue removal).

### Raman spectrometry

#### Surface-enhanced Raman spectrometry of genomic DNA

**Figure 1:**
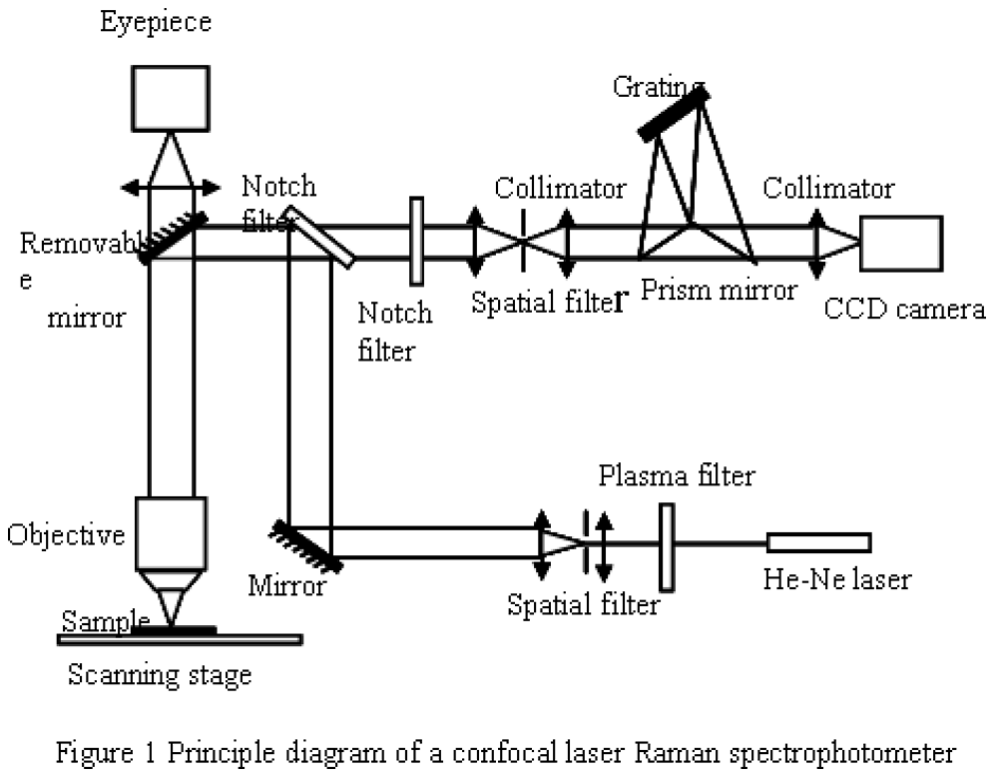
Principle diagram of a confocal laser Raman spectrophotometer.

RENISHAW confocal Raman spectrometry was used with a He-Ne laser. The excitation wavelength was 632.8 nm, and the power was 5 mW. The integration time was 10 s×3, and the resolution was 1 cm^-1^. Twenty microliters of DNA solution was loaded on each slide, and 20 μl of DNA solution from cancer cells was loaded on an enhanced matrix. The Raman spectrum was then analyzed. The scanning range was 400–2000 cm^-1^. The principle for confocal Raman spectrometry is illustrated in Figure 1. During the examination, the sample was placed at the focal plane of the objective. The excitation laser was focused through the objective and then focused on the sample. The excited sample emitted Raman scattered light, which passed through the observation lens and the grating and was ultimately collected by a charge-coupled device (CCD) to generate the Raman spectrum.

#### Raman spectrometry of nuclei

A confocal Raman spectrometer (ThermoFisher) was used. The instrument parameters were same as those described in 2.2.5.1. A 100x objective was used to observe the sample. Representative nuclei on H&E-stained slides were examined using Raman spectrometry.

#### Raman spectrometry of tissue

Tissue was removed from the storage vial and thawed at room temperature. The tissue was then spread and placed on a glass slide. The tissue was examined under a RENISHAW confocal Raman spectrophotometer with a He-Ne laser, an excitation wavelength of 785 nm, a power of 30 mW, an integration time of 10 s x 3, a resolution of 1 cm^-1^, a range of 400–2000 cm^-1^, and a 100x objective. Each specimen was measured under the same condition. Three observation fields were randomly selected from each tissue sample. The average was used to represent the Raman spectrum of the sample. Fifteen normal tissues (from 15 healthy individuals) and 15 gastric cancer tissues (from 15 gastric cancer patients) were examined using Raman spectrometry. After measurement, tissues were fixed with 10% formalin and then been pathological confirmed.

### Data management

All data were normalized, and intensity was standardized. Basal level background was subtracted. Data were analyzed using the following software packages: NGSLabSpec, Microsoft Excel, Origin, Graphpad Prism and IBM SPSS. Search of Characteristic peaks was completed with NGSLabSpec and the parameter setting was kept consistant during the whole searching process.

### Statistical analysis of tissues

Average spectrum of 15 normal and cancerous gastric tissues were calculated respectively. And the ratio of relative peak intensity were also calculated. Two Independent Sample t-Test was used to analyze the ratio of relative peak intensity between normal and cancer by IBM SPSS (P<0.05 means there is significant difference between groups). Meanwhile, the accuracy, sensitivity and specificity were calculated for ratio in discriminating cancer from normal. The Receiver Operating Characteristic curve (ROC Curve) was draw by Graphpad Prism. At the same time, the average raman shift of Characteristic peaks was calculated. Scatter diagram was drawed to display the distribution of Characteristic peaks.

Attributable Raman bands are displayed in Table 1 [1]–[10], [13]–[25].

**Table 1:** Tentative assignments of Raman bands (human tissue).

Position of character peak	Biochemical Assignments	Biomolecular Assignments
622	δc-c (Twisted) phenylalanine	Phenylalanine, Tyrosine
645	dC	Nucleic acid
669	dT	Nucleic acid
721	dA	Nucleic acid
758	*ν*s (Indole ring breathing)	Tryptophan
786	*ν*s_PO2-_ group	DNA, RNA
829	*ν*as_PO2-_	Nucleic acid
855	*ν*_ C-C_ Proline	Protein (collagen)
877	*ν*_C-C_ Hydroxyproline	Protein (collagen)
938	*ν*_C-C_ Proline and valine (α-helix)	Protein (collagen)
957	δ_CH3_ (deformed)	Lipid, protein
1001–1004	*ν* ring breathing	Phenylalanine, protein
1033	δ_C-H_ (Plane bending) aromatic compound	Phenylalanine, tryptophan, tyrosine
1065	*ν*_-C = C = C_-	
1083–1095	*ν*s _-O-P-O-_, v _C-C_ phospholipid	Nucleic acid (DNA, RNA)
1127	*ν*_C-N_ protein, v _C-C_ lipid	Protein, Lipid
1157.00	Polyene chain	Carotenoids
1173	δ_C-H_ (In-plane bending) phenylalanine, tyrosine	Phenylalanine, tyrosine (protein)
1209	*ν*_C-C6H5_ Tryptophan and phenylalanine	Tryptophan, phenylalanine (protein)
1230–1240	Amino compound III (β fold)	
1245–1255	Amino compound III (random coil, corner)	
1264–1272	Amino compound III (α-helix)	
1266	*ν*_C-H_ and δ_H-N-_ (Bending) amino compound III	Protein
1288–1304	Lipid ch2 bending vibration and bending vibration ch2ch3	
1320–1340	*ν*_Ch2ch3_ and δ_Ch2ch3_ (Swing) proteins and nucleic acids	Protein, nucleic acid
	δ_C-H_ (Plane deformation) ordinary olefin	Unsaturated fatty acid
1448	δ_CH2_ (Bending) proteins and lipids	Protein, Lipid
1527	*ν*_C-C_ Carotenoids	Carotenoid
1551	*ν*as _-NO2_	
1585	*ν* c = c Lipid	Unsaturated fatty acid
1605	*ν*_C = C_ Aromatic compound	Phenylalanine, tyrosine
1617	*ν*_C = C_	Porphyrin and tryptophan
1640-1680	Amino compounds I, α helix	Protein

*ν*: stretching vibration, *νa*s: asymmetric stretching vibration, *ν*s: symmetric stretching vibration, δ: bending, deformed, swing (relative peak intensity = the peak intensity/average intensity of the full spectrum).

## Results

### Raman spectra of genomic DNA of normal gastric mucosa and gastric cancer

**Figure 2:**
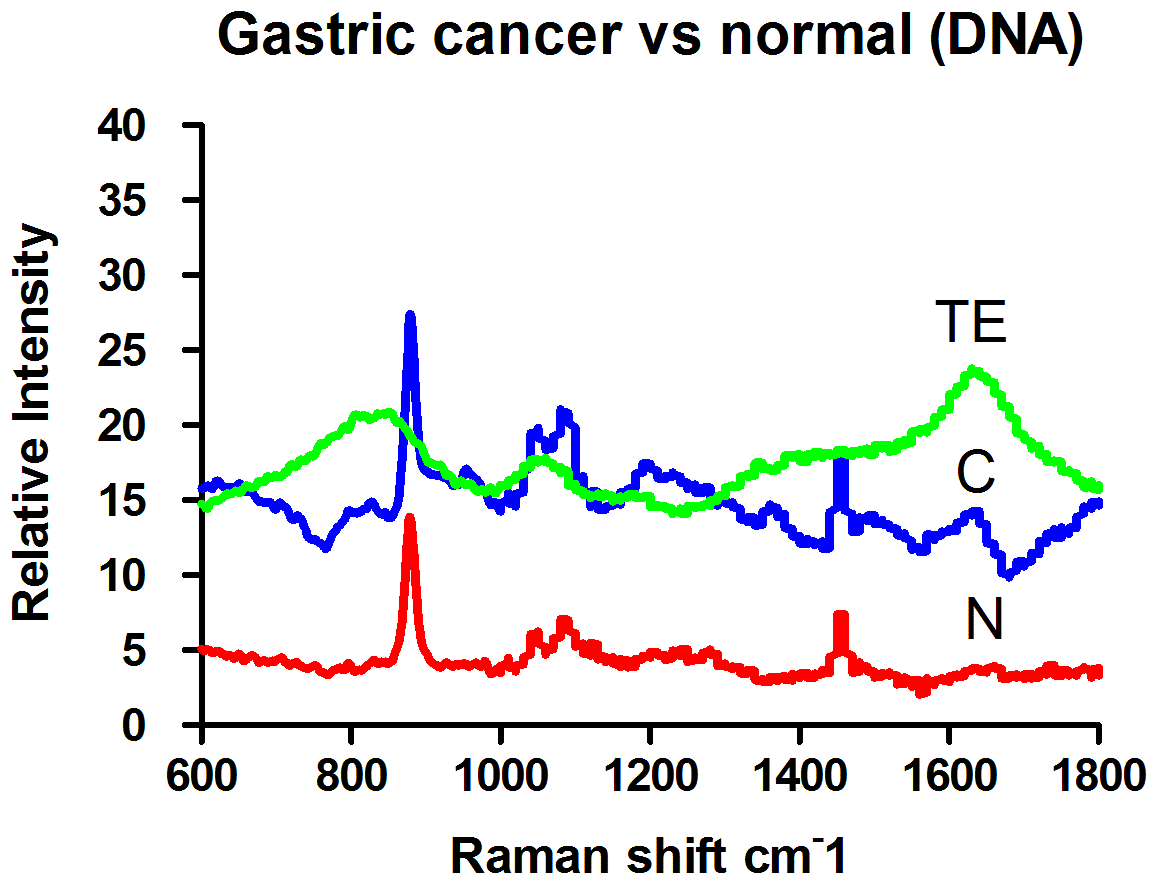
The Raman spectrum of gastric mucosal tissue DNA (Normal tissue: N. Gastric cancer tissue: C. Elution buffer: TE).

**Figure 3:**
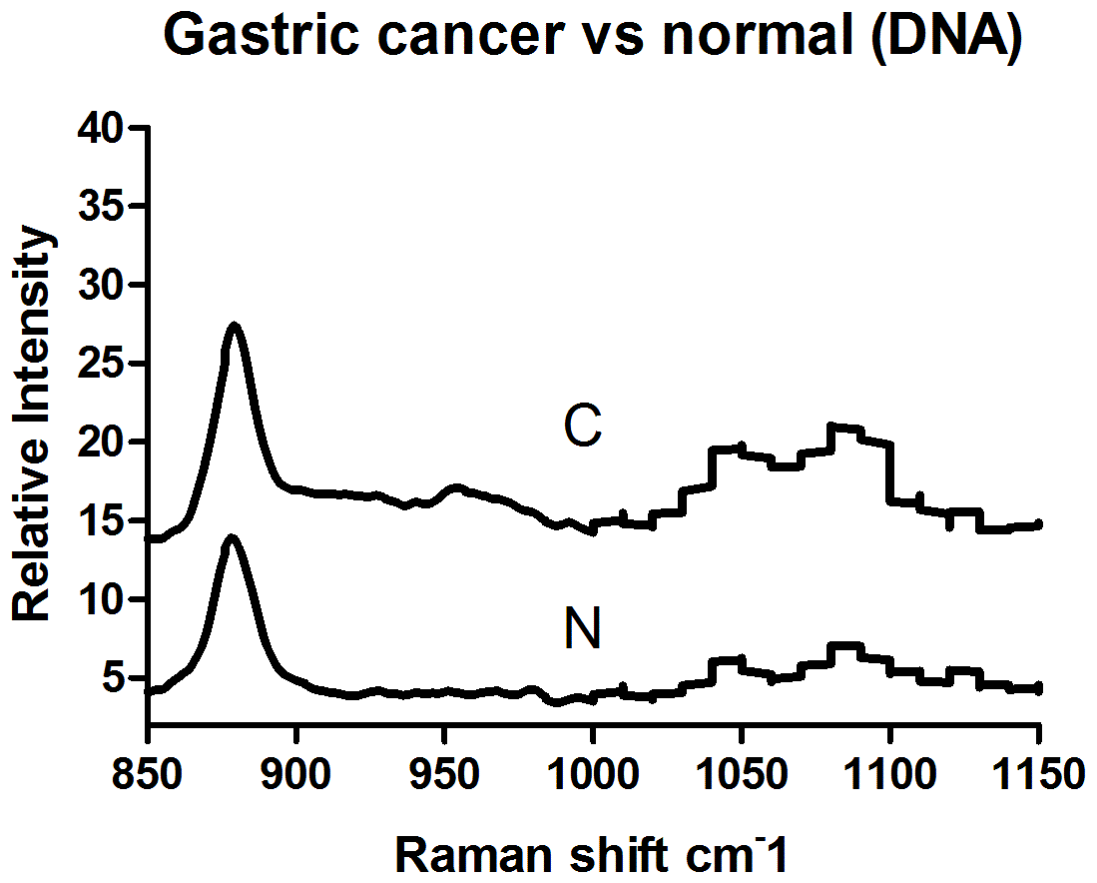
The Raman spectrum of gastric mucosal tissue DNA (Normal tissue: N Gastric cancer tissue: C).

The Raman spectra of genomic DNA from normal gastric mucosa (N) and gastric cancer (C) are illustrated in Figure 2. Line TE represents the Raman spectrum of the elution buffer TE used for DNA extraction. The Raman spectrum of TE showed wide and gentle peaks, indicating weak Raman light scattering. The effects of TE on experiments were easily removed. The Raman spectrum of genomic DNA was simple. The Raman spectrum of gastric cancer DNA exhibited changes at 950 cm^-1^, 1010 cm^-1^, 1050 cm^-1^, 1090 cm^-1^, and 1100–1600 cm^-1^. An extra peak appeared at 950 cm^-1^. The intensity of the peaks at 1010 cm^-1^ and 1050 cm^-1^ (I_1050 cm-1_/I_1010 cm-1_) increased. Twin peaks appeared at 1090 cm^-1^. Between 1100 and 1600 cm^-1^ on the spectrum of cancer DNA, vibration peaks with significant relative intensity appeared at 1213 cm^-1^ and 1374 cm^-1^, which were absent in the spectrum of normal DNA. To present the results with better clarity, we have displayed an enlarged view of the spectrum between 850 and 1150 cm^-1^ in Figure 3.

### The Raman spectra of nuclei of normal gastric mucosa and gastric cancer

**Figure 4:**
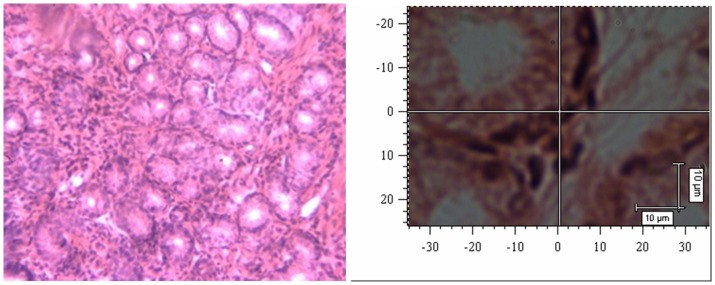
Normal mucosal tissue (H&E 200x). 4-2 Confocal Raman microscopy image of a normal mucosal tissue section.

**Figure 5:**
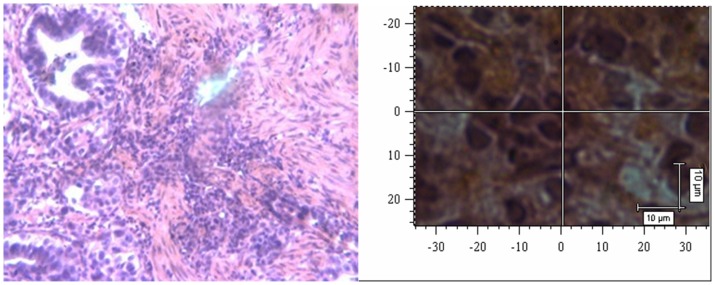
Gastric cancer tissue (H&E 200x). Figure 5-2 Confocal Raman microscopy image of a gastric cancer tissue section.

**Figure 6:**
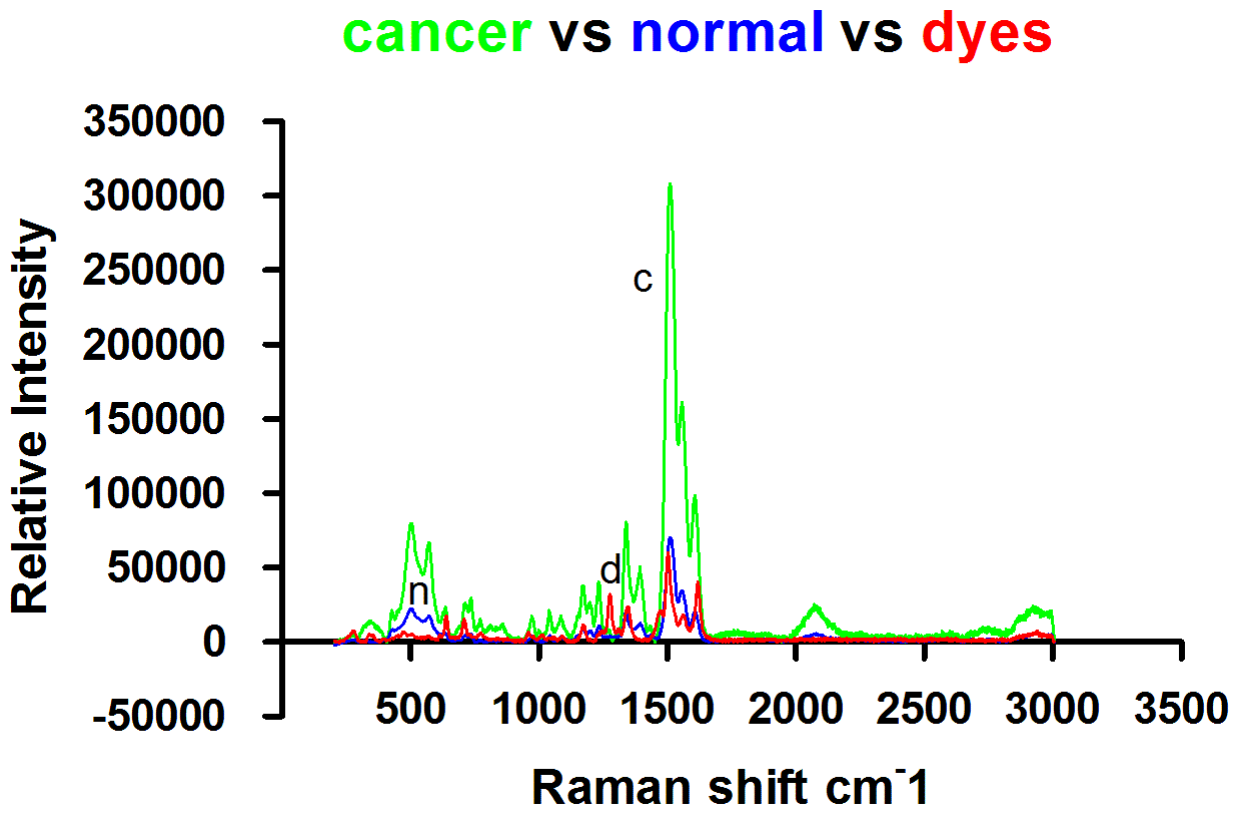
Raman spectra of nuclei from mucosal sections (Normal: n. Cancer: c. H&E dyes: d).

Nuclei were visualized by standard optical microscopy or confocal Raman spectrophotometry on H&E-stained slides, and representative images are displayed in Figure 4-1 and 4-2 (normal mucosal cells) and in Figure 5-1 and 5-2 (gastric cancer cells). The Raman spectra of nuclei are illustrated in Figure 6; N represents the Raman spectrum of normal mucosal nuclei, and C represents the Raman spectrum of gastric cancer nuclei. The H&E dyes exhibited multiple peaks at 471 cm^-1^, 704 cm^-1^, and 774 cm^-1^, some of which overlapped with the Raman peaks representing nuclei, such as the peak at 1344 cm^-1^. Thus, the peaks of the H&E dyes could not be easily removed and affected the Raman spectra of the tissue to some degree. Nevertheless, significant differences in the intensity, position, and number of signature peaks in the Raman spectra between normal and cancer nuclei were detected. The positions of the peaks at 505 cm^-1^, 755 cm^-1^, 1557 cm^-1^, and 1607 cm^-1^ remained unchanged, indicating that instrument calibration prior to the measurement was accurate and that the shift of the signature peaks in a Raman spectrum is significant. The intensity of the peak representing nucleic acids in cancer cell nuclei at 1085 cm^-1^ was increased, and the position of the peak also shifted to 1087 cm^-1^. The relative intensity of the signature peaks representing amino acids (proteins) at 755 cm^-1^ and 1607 cm^-1^ was increased in cancer cell nuclei compared with normal cell nuclei. The relative intensity of the signature peak representing amino compound III at 1233 cm^-1^ was reduced, and the position shifted to 1231 cm^-1^ in cancer cell nuclei. In addition, the signature peak representing amino compound III at 1262 cm^-1^ disappeared in cancer cell nuclei but remained in normal cell nuclei. The distribution of signature peaks is listed in Table 2.

**Table 2:** The distribution of signature peaks in the Raman spectra of nuclei from H&E-stained sections.

Gastric cancer cell nuclei (cm^-1^)	Normal mucosal cell nuclei (cm^-1^)	Ratio of relative intensity (cancer/normal)	H&E dyes (cm^-1^)
505	505	4.27/5.01	471.63
755	755	0.51/0.05	639.62
	974		709.58
1040	1043	1.15/1.03	774.69
1087	1085	0.96/0.80	958.16
1171	1173	2.03/2.06	1171.33
1199	1198	1.43/1.67	1275.72
1231	1233	2.18/2.52	1311.70
	1262		1343.71
	1298		1470.10
1339	1342	4.33/4.70	1502.20
1557	1557	8.65/7.75	1560.45
1607	1607	5.28/4.63	1619.04

### Raman spectra of normal mucosal tissue and gastric cancer tissue

**Figure 7:**
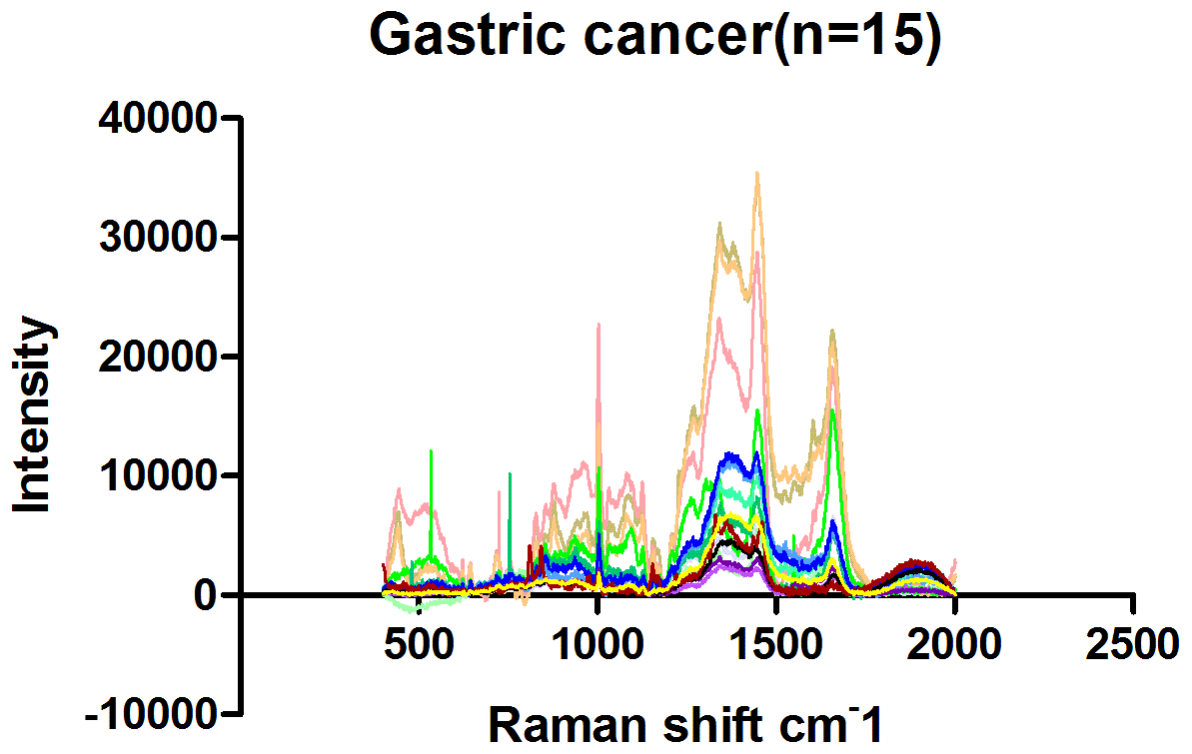
Raman spectra of 15 gastric cancer tissues.

**Figure 8:**
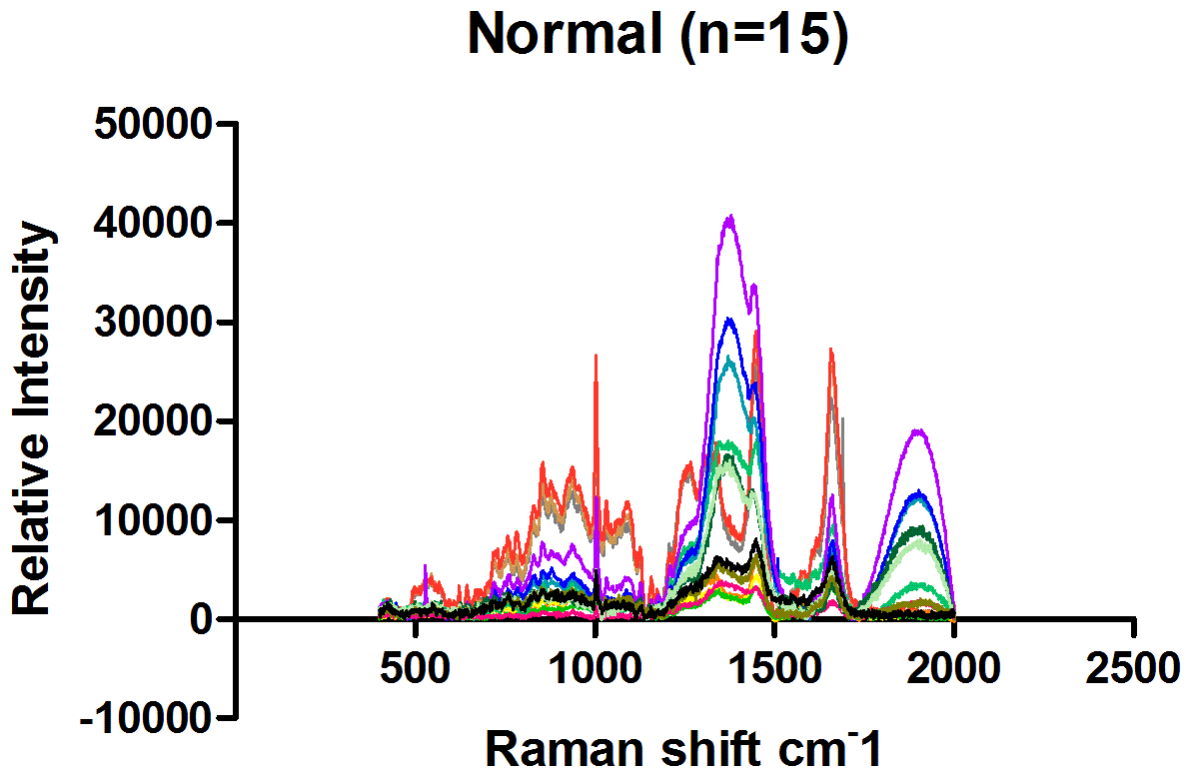
Raman spectra of 15 normal mucosal tissues.

**Figure 9:**
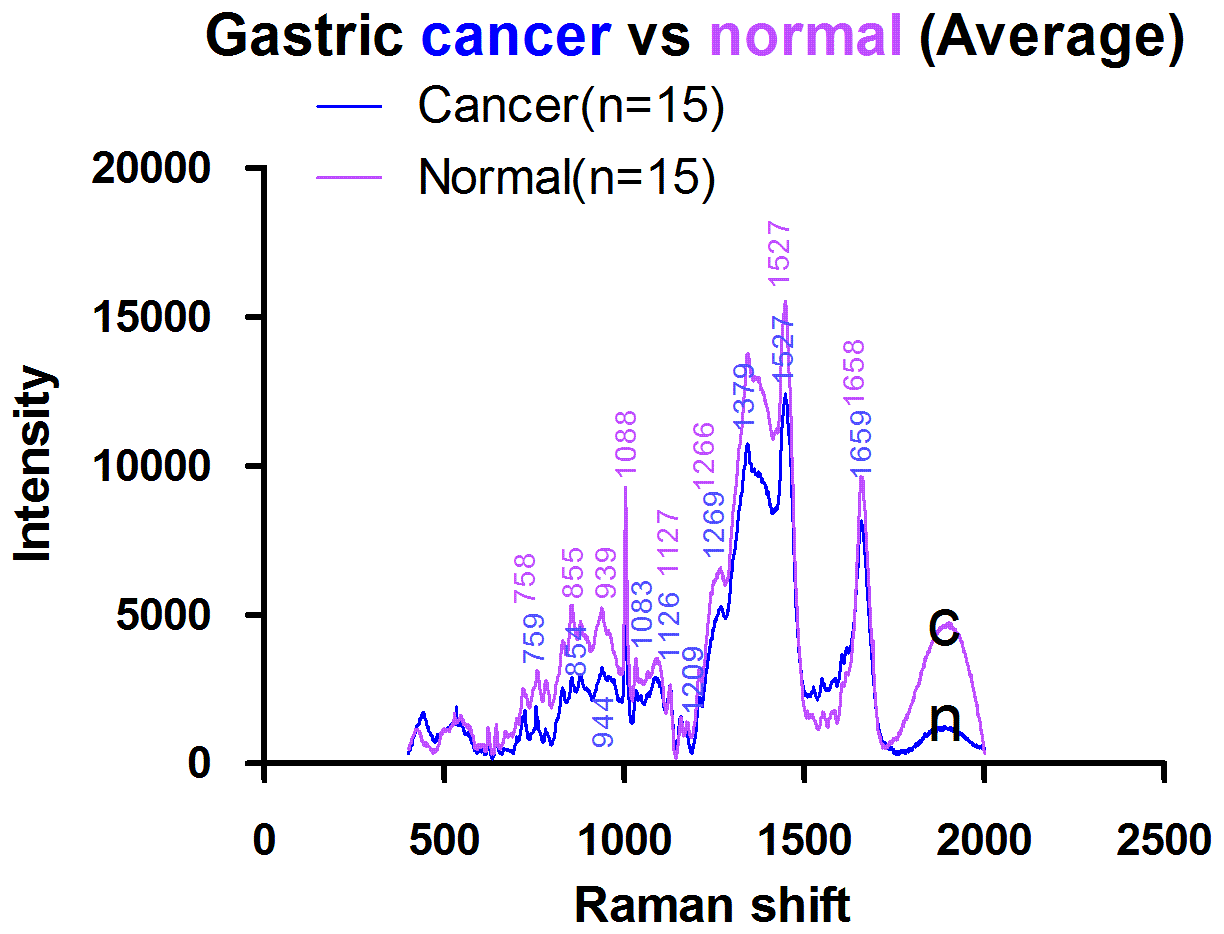
Average Raman spectra of mucosal tissues (Normal: n. Gastric cancer: c).

**Figure 10:**
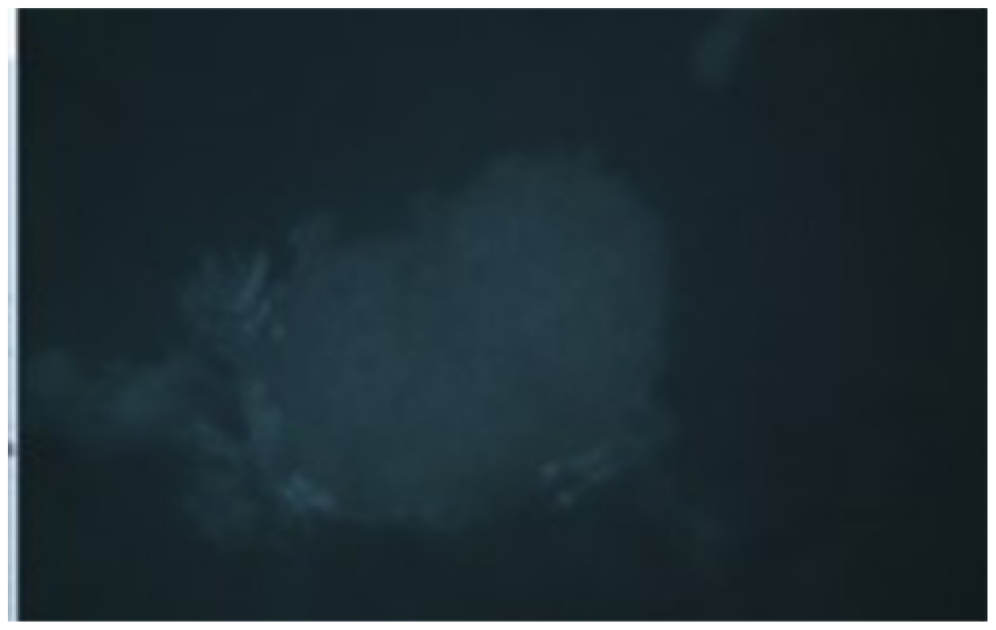
Image of tissue obtained by confocal Raman spectrometry (100x).

**Figure 11:**
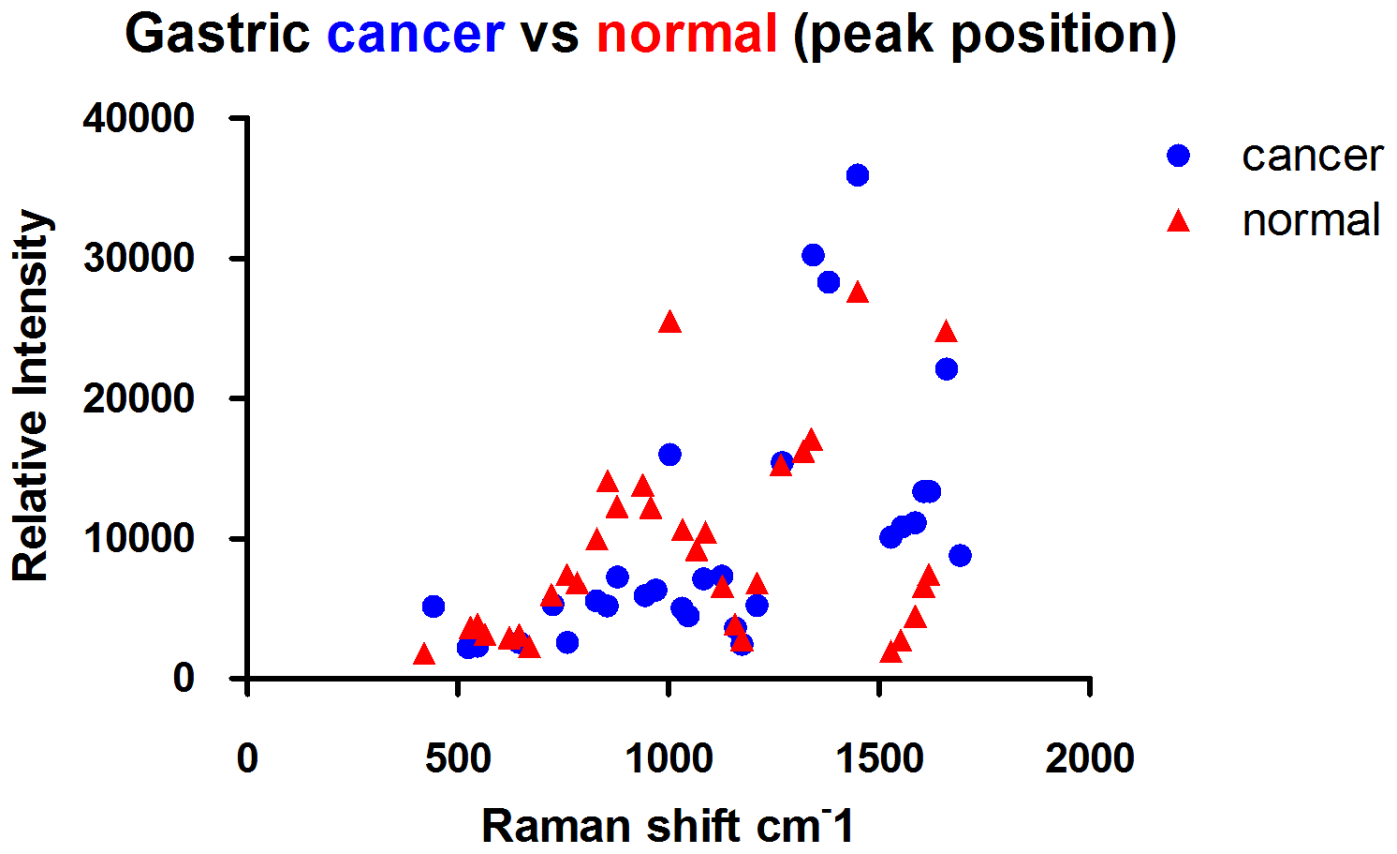
Distribution of signature peaks of gastric cancer and normal tissue.

The full Raman spectra of normal and cancer tissue are illustrated in Figures 7 and 8. Figure 9 shows the average Raman spectra of normal mucosal tissue and cancer tissue. Figure 10 displays the image of tissue obtained by confocal Raman spectrophotometry. Normal and cancer tissues exhibited significant differences in the position, relative intensity, shape, and number of signature peaks in their Raman spectra. The positions of the peaks at 645 cm^-1^, 1003 cm^-1^, 1173 cm^-1^, 1209 cm^-1^, 1448 cm^-1^, 1527 cm^-1^, and 1585 cm^-1^ remained unchanged, suggesting that instrument calibration prior to the experiment was accurate, and the possibility that measurement errors and environment factors caused peak shifts can be excluded. Compared with normal tissue, the position of the peaks at 758 cm^-1^, 854 cm^-1^, 876 cm^-1^, 938 cm^-1^, 1087 cm^-1^, 1033 cm^-1^,1266 cm^-1^, 1338 cm^-1^, 1617 cm^-1^, and 1658 cm^-1^ shifted significantly in cancer tissue. The shifts ranged between 1 to 5 cm^-1^ and the average shift was 2.31±1.62 cm^-1^. Between 1338 cm^-1^ and 1447 cm^-1^, the spectrum of normal tissue appeared as an apparent dip without a peak, although a peak appeared at 1379 cm^-1^ in the spectrum of cancer tissue. The relative intensities of I_1685 cm-1_, I_1209 cm-1_, I_1126 cm-1_, and I_1266 cm-1_ (1269 cm^-1^) did not increased or decreased obviously in cancer tissue compared with normal tissue while I_1585 cm-1_ and I_1527 cm-1_ were significantly larger than in normal tissue. It is recognized that the detection of non-aromatic amino acids is challenging because they produce weak Raman vibration signals due to weak polarity. However, aromatic amino acids can exhibit obvious signature peaks in a Raman spectrum due to the vibration of benzene ring. The distribution of signature peaks in the Raman spectra of normal and cancer tissue are listed in Table 3 and are also distinctly showed by scatter diagram in Figure 11. According to Table 1, we found that the signature peaks in the spectrum of cancer tissue represent macromolecules, such as proteins, nucleic acids, and lipids, indicating that the biochemical composition undergoes changes in cancer tissue.

**Table 3:** Distribution of Raman peaks of tissues.

Gastric cancer (cm^-1^)	Normal (cm^-1^)	Relative intensity (Cancer/Normal)
	622	
645	645	0.37±0.08/0.35±0.16
	669	
725	721	0.67±0.31/0.65±0.28
759	758	0.77±0.42/0.84±0.34
	783	
828	829	1.65±1.25/1.06±0.38
854	855	1.23±0.47/1.34±0.57
878	877	1.11±0.41/1.18±0.54
944	938	1.20±0.47/1.35±0.62
963	963	1.05±0.44/1.11±0.54
969	957	1.00±0.41/1.15±0.56
1003	1003	2.2±0.72/2.31±1.15
1032	1033	0.84±0.36/0.90±0.55
	1066	
1083	1088	0.93±0.40/0.92±0.50
1126	1127	0.79±0.38/0.71±0.30
1158	1157	0.54±0.26/0.42±0.18
1173	1173	0.39±0.15/0.39±0.14
1209	1209	0.79±0.19/0.88±0.21
1269	1266	1.74±0.39/1.72±0.50
1343	1338	3.79±0.47/3.44±0.64
1379		
1448	1448	4.20±0.58/3.99±0.38
1527	1527	0.80±0.54/0.42±0.31
1554	1551	0.85±0.48/0.57±0.30
1585	1585	0.88±0.40/0.56±0.37
1605	1605	1.13±0.50/0.83±0.51
1619	1617	1.24±0.49/0.96±0.61
1659	1658	2.81±1.12/2.72±1.29
1692		

**Figure 12:**
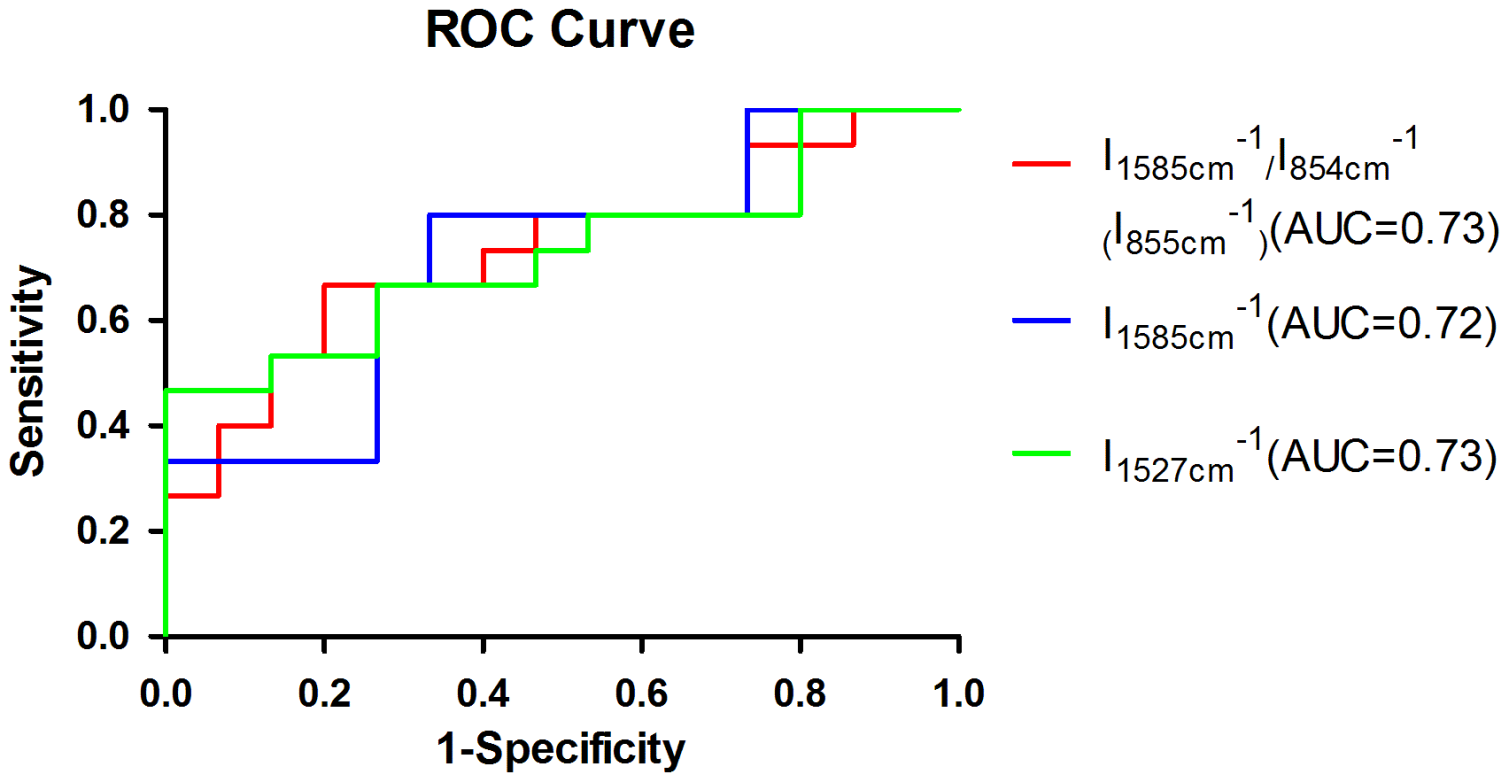
ROC curve from the ratio of relative peak intensity (Two Independent Sample t-Test).

Two Independent Sample t-Test was applied to compare the ratio of relative peak intensity between normal and cancer tissues. And the results showed that I_1585 cm-1_/I_854 cm-1(855 cm-1)_,I_1585 cm-1_ and I_1527 cm-1_ were definitely different between normal and cancer tissues. The accuracy, sensitivity and specificity were showed in Table 4 and ROC curve in Figure 12.

**Table 4:** Ratio of relative peak intensity (Two Independent Sample t-Test).

Ratio of relative peak intensity	Mean	N	P value	Cancer	Normal	Accuracy	Sensitivity	Specificity
I_1585cm-1_	Cancer:0.88±0.44	Cancer:15	0.04	≥0.72	<0.72	73.3%	80%	67%
	Normal:0.56±0.37	Normal:15						
I_1527cm-1_	Cancer:0.80±0.54	Cancer:15	0.03	≥0.40	<0.40	66.7%	67%	73%
	Normal:0.42±0.31	Normal:15						
I_1585cm-1_/I_853cm-1(854cm-1)_	Cancer:0.90±0.74	Cancer:15	0.03	≥0.64	<0.64	73.3%	67%	80%
	Normal:0.42±0.29	Normal:15						

## Discussion

Changes in the nucleus initiate phenotypic changes in tissue and cells. Genomic materials in the nucleus regulate protein synthesis and metabolism in the cytoplasm and extracellular matrix. The most obvious change in cancer cells is that due to excessive DNA replication, nuclei exhibit enlargement to various sizes, deformity, thickening of the nuclear membrane, an increase in nuclear chromatin, condensation of granules, and disproportion of nucleoplasm. For example, it has been reported that during malignant transformation, the extracellular matrix scaffold structure is damaged and microtubules are disassembled, leading to the increase in cancer cell mobility; cancer cells secret enzymes to degrade matrix components and facilitate metastasis. The Raman spectra of nuclei and tissues are composed of the Raman spectra of nucleic acids, proteins, and lipids. The Raman peaks of nucleic acids are mainly produced by the vibration of bases and the DNA backbone, which can be easily masked by signals from other molecules in normal tissue. However, during malignant transformation, cells proliferate in an uncontrolled manner, and intracellular DNA content is significantly increased, which is accompanied by substantial changes in phosphates, deoxyribose, or bases. The Raman spectra of proteins contain information regarding amino acid side chains and are crucial for investigating the interaction between protein structure and function. The Raman signals of lipids are mainly produced by the vibration of the cell membrane, the C-C and C-H bonds of lipids, and C = C of unsaturated fatty acids. We investigated the Raman spectra of the DNA, nuclei, and tissues of gastric cancer and performed differential analysis to reveal changes in macromolecules, their interactions, and the biochemical characteristics of malignant cells and tissues.

### Analysis of Raman spectra of genomic DNA of normal gastric mucosal and cancer tissue

The structural changes in DNA are mainly caused by alterations in phosphates and deoxyribose or bases. A DNA Raman spectrum shows that changes in DNA molecular structure can produce a corresponding specific spectrum. Our results suggest that peaks appearing between 800 and 900 cm^-1^ are produced by the vibration of deoxyribose, which is also called ring-breathing vibration. Ring structure is usually very stable. The intensity of ring-breathing vibration can be used as a reference for the intensity of the DNA Raman spectra of normal mucosal and cancer tissues. Both normal and cancer tissue showed a strong vibration at 878 cm^-1^, and the frequency was consistent.

The peak at 950 cm^-1^ is attributed to deoxyribose vibration and appeared as a weak peak in the cancer DNA spectrum but was absent in normal tissue. The polarity of deoxyribose in cancer genomic DNA undergoes changes during malignant transformation, resulting in the stimulation of a new vibration pattern [26]. Peaks at 1010 cm^-1^ and 1050 cm^-1^ are attributed to the vibration of the C = O bond in the deoxyribose backbone and appeared as strong peaks in both normal and cancer genomic DNA spectra. The positions of the peaks were consistent in the two DNA samples. However, I_1050 cm-1_/I_1010 cm-1_ was larger in cancer genomic DNA than in normal DNA, further suggesting that the polarity of deoxyribose changes during malignant transformation.

The peak at 1090 cm^-1^ representing the vibration of phosphates split into two peaks at 1080 cm^-1^ and 1090 cm^-1^, and the relative intensity of the peak at 1090 cm^-1^ was reduced in cancer genomic DNA. These results indicate that cancer genomic DNA is fragmented. The peaks at 1050 cm^-1^ and 1090 cm^-1^ were significant. The relative intensity of the peak attributed to phosphate vibration (1090 cm^-1^) was higher than that of the peak representing deoxyribose vibration (1050 cm^-1^) in normal tissue, and the phosphate backbone was consistent in normal DNA, indicating stability. In cancer tissue, the intensity of the peak attributed to phosphate vibration was higher than that of the peak representing deoxyribose vibration, suggesting that the phosphate backbone forms stable structures after DNA breakage.

Thus, the DNA of normal mucosal tissues has a stable phosphate backbone, whereas the Raman spectrum of DNA from cancer tissues showed two peaks, one at 1090 cm^-1^ with a higher intensity than the peak at 1050 cm^-1^, indicating that DNA could form a stable phosphate backbone after breakage. The peaks at 950 cm^-1^, 1010 cm^-1^, and 1100-1600 cm^-1^ in the cancer DNA spectrum changed greatly compared with the normal DNA spectrum, suggesting that deoxyribose and bases undergo corresponding structural changes due to DNA breakage.

### Analysis of the Raman spectra of nuclei from normal gastric mucosal and cancer tissue

Our spectral analysis showed that despite the large interference of H&E dyes, we were able to identify a significant difference in the Raman spectra between normal nuclei and cancer nuclei. Peaks at 472 cm^-1^, 710 cm^-1^, and 1171 cm^-1^ were attributed to H&E dyes and absent in the Raman spectra of nuclei. The peak at 1088 cm^-1^ in the spectra of nuclei is attributed to the symmetric stretching vibration of PO^2-^ in nucleic acids. The conformation of the peak at 1088 cm^-1^ was not sensitive. In the Raman spectra of cancer nuclei, this peak shifted to 1084 cm^-1^ and underwent “red shift” (a shift toward low frequency and low vibrational energy; “blue shift” is the opposite), suggesting that DNA single and double strand breakage occurred. This is consistent with our findings in the DNA spectra.

The feature peak at 755 cm^-1^ is attributed to the symmetric stretching vibration of the indole ring in tryptophan. The peak at 1607 cm^-1^ is attributed to the symmetric stretching vibration of C-C in the benzenes of phenylalanine and tyrosine. The relative intensity of these two feature peaks was significantly increased in cancer nuclei, indicating that the protein content in cancer nuclei is increased. Tyrosine residues within histones, the major type of protein inside the nucleus, are targets of phosphorylation. It is known that the ratio of histones to DNA is 1∶1 in chromatin. Thus, the increase in histone content further suggests that active mitosis in cancer nuclei leads to a significant increase in DNA content.

Peaks attributed to amino compound III are mainly produced by C-N stretching vibration and N-H banding vibration and are located at 1230–1300 cm^-1^; these peaks indicate protein β-sheet structure. The spectra from our study showed that in cancer nuclei, the relative intensity of the signature peak attributed to amino compound III at 1233 cm^-1^ was reduced, and the position shifted to 1231 cm^-1^. A peak at 1262 cm^-1^ attributed to amino compound III appeared in the Raman spectra of normal nuclei but was absent in the spectra of cancer nuclei. It is known that the secondary structure of histones is mainly an α helix, and random coils and β sheets predominantly exist in fibrin, suggesting that the content of nuclear matrix proteins, such as fibrin mesh and nuclear lamina, is reduced or that the structure of the nuclear matrix becomes loose in cancer cells. Nuclear lamina fibrins are disassembled at an early stage of mitosis and dissociate from chromatin, promoting chromosome formation. Our results are consistent with this. The other possibility is that the spatial distance between nuclear matrix proteins associated with mitotic nuclear swelling is increased, weakening the chemical bond governing the internal interactions.

We speculate that DNA single- and double-stranded breaks occur in cancer nuclei, the content of nucleic acids and histones is increased, the content of non-histone proteins such as fibrin is reduced, and the structure of non-histone proteins may be loose.

### Analysis of Raman spectra of normal mucosal and cancer tissues

Compared with the Raman spectra of DNA and nuclei, the spectrum of unprocessed cells in tissue contained richer information. Compared with normal tissue, the peak representing the symmetric stretching vibration of PO^2-^ in nucleic acids shifted from 1088 cm^-1^ to 1083 cm^-1^ in cancer tissues; “red shift” occurred. These results indicate that the length of the phosphodiester bonds in the nucleic acids of cancer tissues is elongated and that the vibration of interacting bonds is weakened, suggesting that the nucleic acid backbone structure is loose in cancer cells.

The peak at 1527 cm^-1^ is attributed to carotenoid [14]. We analyzed I_1527cm-1_ between normal and malignant gastric tissues with Two Independent Sample t-Test and then discovered that the relative intensity of the peak I_1527 cm-1_ was significantly stronger in cancer tissue than in normal tissue (p<0.05), indicating that carotenoid content in cancer tissue is increased. Carotenoid consisted of carotene, carotol, propane diacid and so on. Human tissues contained carotene mainly, includingβ- carotene, α- carotene,γ- carotene and so on. β- carotene is an antioxidant that can protect proteins and nucleic acids from damage by free radicals and reduce the damage to genetic material and the cell membrane. The significant increase of Carotenoid in cancer cells suggests that cancer cells probably evolve an enhanced capability to resist damage. In addition to its antioxidant function, carotenoid is involved in the synthesis of glycoproteins in vivo. The proliferation and differentiation of normal gastrointestinal epithelial cells require retinoic acid, indicating that cancer cells might need to synthesize more glycoproteins than normal cells and that metabolism in cancer cells may be more active than in normal cells.

Compared with normal tissue, the peak representing the ring-breathing vibration of the indole ring of tryptophan in the cancer tissue spectrum shifted from 758 cm^-1^ to 759 cm^-1^, “blue shift” occured. These results suggest that the structure of tryptophan is more stable in cancer tissue or its increased stabilizition was affected by the activation of the neighboring functional groups. It may indicates that more tryptophan is located in a hydrophobic environment, such as the core of globin [20]. Our results also indicate that the variety of protein species and the conformation of proteins are changed in cancer tissues.

In the spectrum of cancer tissue between 1338 and 1447 cm^-1^, a peak representing unsaturated fatty acids appeared at 1379 cm^-1^ that was absent in the spectrum of normal tissue. The relative intensity of the peak representing unsaturated fatty acids at 1585 cm^-1^, I_1585 cm-1_, was significantly increased in cancer tissue compared with normal tissues (Two Independent sample t-Test, p<0.05), suggesting that the content of unsaturated fatty acids in cancer tissue is increased. Cell membrane mobility is positively correlated with the content of fatty acids in cells. The increase of unsaturated fatty acid content in cancer cells suggests that cancer cell membrane mobility increases, which facilitates the transportation and metabolism of transmembrane molecules.

Compared with normal tissue, the peak at 938 cm^-1^ shifted to 944 cm^-1^ in cancer tissue, a “blue shift”, indicating that the energy of vibration increased. This peak is attributed to the stretching vibrations of proline and valine [24] and represents the α helix of collagen. This result indicates a conformational change in collagen structure in cancer tissue; factors contributing to a peak shift include activation, adhesion, and twisting of functional groups. More of the α helix might be exposed, activated, and formed to enhance the vibration. However, the relative intensity of I_1585cm-1_/_I853cm-(854 cm-1)_ in cancer tissues was significantly stronger than that of normal ones (Two Independent sample t-Test, p<0.05), indicating that collagen content in cancer tissue is significantly reduced. Cancer cells synthesize and secrete matrix metalloproteinases to degrade matrix proteins such as collagen, facilitating cancer metastasis. It is also possible that epithelium thickening caused by cancer cell proliferation masks the Raman signal of collagen in the matrix [22].

The Raman peaks at 1658 cm^-1^, 1033 cm^-1^, 1266 cm^-1^and 1127 cm^-1^ represent proteins [4]-[6], [13], [20]. Compared with normal tissue, the position of 1658 cm^-1^,1127 cm^-1^, 1033 cm^-1^ and 1266 cm^-1^were shifted in cancer tissue to various degrees, suggesting that the interactions between chemical bonds of amino acids are weakened in cancer cells. For example, hydrogen bonds might be damaged, resulting in a loose and random protein structure or changes in the microenvironment of amino acid residues, such as increases in the assembly or disassembly of α helices and β sheets. The peaks at 1266 cm^-1^ and 1658 cm^-1^ represent the α helices of histones [20] and were shifted to 1269 cm^-1^ and 1659 cm^-1^ in cancer tissue. Histones are rich in basic amino acids, carry positive charges, and bind DNA carrying negative charges to inhibit DNA replication and transcription. After histones are phosphorylated or acetylated, the histone charge is reduced, leading to weak DNA binding and promoting replication and transcription. The vibration of histones in cancer tissue showed “blue shift”, suggesting that the degree of phosphorylation on the serine, tyrosine and lysine residues of the histones may be increased, which would lead to decreased histone charge, increased vibration energy, and reduced histone-DNA binding.

### Comparative analysis of the Raman spectra of DNA, nuclei, and tissue

The results of the comparative analysis of the Raman spectra of genomic DNA, nuclei, and tissue demonstrated that genomic DNA Raman peaks are relatively simple and that the Raman signature peaks of tissue contain rich information. The Raman spectra of tissue contain information regarding nuclei, cytoplasm, and the extracellular matrix. In addition, complex information about macromolecules such as proteins and lipids can be revealed from unprocessed tissue.

The peak at 1088 cm^-1^ representing the nucleic acid phosphate backbone shifted in the spectra of the genomic DNA, nuclei, and tissue of gastric cancer compared with normal tissue. The peak showed “redshift” in the Raman spectra of genomic DNA and tissue, suggesting that internal chemical bonds are not consistent, resulting in increased vibration patterns and decreased vibration energy. These results indicate that the nucleic acid phosphate backbone in cancer cells is unstable and that DNA double strand breakage may occur. Re-establishment of a relatively stable backbone may occur after DNA breakage. However, this peak exhibited “blue shift” in the Raman spectra of nuclei on H&E slides. This phenomenon might be caused by the fact that the binding of the basic dye hematoxylin to DNA reduces the positive charges on the DNA, enhancing the interactions among internal chemical bonds and increasing vibration energy. The relative intensity of this peak was increased in the spectra of DNA and nuclei but reduced in the tissue spectrum. This result might be because tissue is rich in proteins and lipids, which could partially mask the signals of nuclei acids.

The Raman spectrum of tissue showed signature peaks attributed to various types of proteins. The content of histones was higher than the non-histone protein content in nuclei, resulting in a relatively simple spectrum. We used Raman spectrometry to examine nuclei and found that histone content is increased in cancer cells. Combined with the Raman spectrum of tissue, we further discovered that histone structure is most likely stable in cancer cells, and this might be associated with a high degree of histone phosphorylation, which reduces charges on the histones.

Compared with the Raman spectrum of tissue, we found that the Raman spectrum of nuclei showed relatively fewer peaks attributed to lipids. Chromatin is composed of DNA and histones. Protein content is approximately 80%, and nuclear matrix proteins are approximately 90% in the nucleolus, but the content of lipids in the nucleus is minimal. Our results further support this conclusion.

In this study, we used Raman spectrometry to investigate the biochemical changes of molecules associated with gastric malignant transformation. Our study not only provides new evidence to support recognized conclusions from a new perspective but also reveals new findings including changes in the environment of tryptophan and alterations in the structure and content of nuclear matrix proteins. But the sample size of our study was constrainted by experimental conditions and maybe not large enough to explore more differences and relevant significance between gastric normal and cancer. So we will enlarge sample size to further investigate these changes in our future studies. Raman peak shift is associated with the structure, symmetry, electronic environment, and chemical bonds of molecules. The in vitro environment of isolated mucosal tissue is markedly different from the in vivo condition because of added effects such as blood flow and gastric acid. There is no doubt that performing real-time Raman spectrometry on tissue in vivo will be our research focus in the future. Our study also provides a basis for the establishment of a Raman spectrum library of mucosal tissue by large sample statistics.

## Conclusions

We used regular Raman spectrometry and surface-enhanced Raman spectrometry to examine the genomic DNA, nuclei, and tissue of normal mucosa and gastric cancer. We comparatively analyzed the Raman spectra to determine the spatial structural changes of macromolecules during gastric cancer initiation and investigated the signature Raman peaks at different stages. Our results demonstrate the following:

1. In the Raman spectra of DNA, nuclei, and tissue, the position of the peak at 1088 cm^-1^ representing the nucleic acid backbone shifted, and the relative intensity of the peak also changed in cancer tissue, indicating that the nucleic acid phosphate backbone is unstable in cancer and that DNA single- and double-strand breakage might occur.

2. Comprehensive analysis of the Raman spectra of nuclei and tissue showed that histone content is increased and that histones are more stable in cancer nuclei.

3. The absence of signature peaks for lipids in the nucleus suggests that nuclei contain trace amount of lipids.

4. Compared with normal mucosal tissue, collagen content is reduced in cancer tissue, suggesting that cancer cells might secret matrix metalloproteinases to degrade collagen and facilitate metastasis. The increase in unsaturated fatty acid content suggests that cancer cell membrane mobility is increased, facilitating transmembrane transportation and cancer cell distal metastasis. The increase in carotenoid content indicates that cancer cells have active metabolism and that more tryptophans are hidden in hydrophobic environments, suggesting changes in protein content, species, and conformations in cancer cells.

Based on the Raman spectra, we are able to discriminate normal versus cancer tissue and observe changes in signature Raman peaks representing structural changes of macromolecules. Our study sheds new light on the application of Raman spectrometry in clinical practice, including uses for real-time diagnosis, early monitoring, pathogenesis investigation, and drug efficacy assessment. We used Raman spectrometry to examine the genomic DNA, nuclei, and tissue of normal mucosal and gastric cancer. Our study provides a novel strategy for investigating the mechanism underlying gastric cancer tumorigenesis and early diagnosis.
